# Successful management of elderly breast cancer patients treated without radiotherapy

**DOI:** 10.1186/1477-7819-5-62

**Published:** 2007-06-03

**Authors:** Kalliope Valassiadou, David AL Morgan, John FR Robertson, Sarah E Pinder, Kwok-Leung Cheung

**Affiliations:** 1 Department of Clinical Oncology, Nottingham City Hospital, UK; 2 Division of Histopathology, University of Nottingham, Nottingham, UK; 3 Division of Breast Surgery, University of Nottingham, Nottingham, UK

## Abstract

**Background:**

Breast cancer in the elderly may follow a less aggressive course. There are data suggesting that radiotherapy (RT) following breast conserving surgery (BCS) for invasive carcinoma may not be necessary in some elderly patients. The addition of RT to surgery might constitute an imposition to such patients due to age-related factors. The aim of this study was to assess the efficacy of BCS without adjuvant RT in this group of patients.

**Patients and methods:**

A retrospective review of 92 elderly (median age 75 years; range: 70 – 87 years) patients (analysed as 93 'patients' due to one patient having bilateral cancers) managed in a dedicated breast clinic and who underwent BCS for invasive carcinoma was carried out. Eighty-three patients did not receive postoperative RT to the breast (no-RT group) whereas the remaining 10 had RT (RT-group).

**Results:**

The median age in this group was 75 (range 70 – 87) years. The mean tumour size was 18 mm with a median follow-up of 37 (range 6 – 142) months. In the no RT group, adjuvant endocrine therapy with tamoxifen was given to 40/53 patients. No patients in the oestrogen receptor (ER) negative group received tamoxifen. The local recurrence (LR) rate in this group was 8.4% (2.4% per year, n = 7/83), with median time to LR of 17 months. In this no-RT group LR was correlated to ER status (2/53 ER+, 5/26ER-, p = 0.024) and margins of excision (n = 1/54 >5 mm, 2/17 1–5 mm, 4/12 <1 mm, p = 0.001). Within the ER positive group the LR rate was 0.92% per annum (0.62% per annum in patients treated with adjuvant tamoxifen, regardless of margin status). Breast cancer specific survival was correlated to histological grade (p < 0.05) and ER status (p < 0.05).

**Conclusion:**

It would appear that omission of RT following successful BCS in elderly patients with ER positive tumours receiving adjuvant tamoxifen may be acceptable. The LR rate as shown in this retrospective study is highly comparable to that of younger patients treated by conventional therapy. This concept is now being evaluated prospectively following a change in treatment practice.

## Background

The majority of breast cancers are diagnosed in women > 65 years of age, although research efforts are, for the most part, focused on younger patients. Treatment of breast cancer in the elderly is controversial and is often extrapolated from their younger counterparts [1]. However, it is evident that there is a huge variation in treatment patterns due to patient age [2-6]. A greater knowledge of breast cancer behaviour and optimal treatment in the elderly is urgently needed. It has been suggested that breast cancer may follow a less aggressive course in older women. This may partly be due to the more frequent detection of breast cancer in earlier stages in this group compared to younger patients where the breast gland is denser. Some data suggest that elderly breast cancers have inherently different biological features and clinical behaviour; for instance, breast cancer in the elderly tends very often to be oestrogen receptor (ER) positive [7]. However, further studies are required to evaluate the benefit from adjuvant treatments in this group of patients.

Adjuvant radiotherapy to the breast after breast conserving surgery (BCS) is the mainstay of treatment to achieve local control in patients with invasive carcinoma. However, the benefit from any adjuvant treatment is directly related to the patients overall co-morbidity and life expectancy. The addition of radiotherapy may constitute an imposition to such patients due to its side effects and other age-related factors such as inconvenience.

This retrospective analysis aimed to address the efficacy of BCS without adjuvant radiotherapy in the elderly population.

## Patients and methods

Since 1973 all elderly (> 70 years) patients with early primary breast cancer (< 5 cm) in Nottingham have been managed in a dedicated Elderly Primary Breast Cancer Clinic according to a defined set of treatment guidelines. During the period of 1989 – 2000, patients with invasive breast carcinoma < 3 cm clinically were given the choice between BCS and mastectomy. Those who had ER positive cancer were also given the option of receiving primary endocrine therapy. Searching the patient database identified 92 patients with primary invasive breast carcinoma over the age of 70 during this period who were treated with BCS as the primary treatment. These patients either had ER positive tumours but did not receive primary endocrine therapy, or had ER negative tumours. Postoperative intact breast irradiation following BCS was not routine for the elderly patients before 1999.

Local recurrence was defined as histologically proven ipsilateral recurrence of breast carcinoma within the treated breast following previous BCS. It was usually located in close proximity to the location of the previously treated primary carcinoma, otherwise histology review to confirm morphological similarity was required. Tumour with a Histochemical score (H-score) of = 50 by standard immunohistochemistry studies was deemed ER positive.

The medical records of the 92 patients were reviewed in order to identify demographic and follow up details, tumour characteristics, local, regional and systemic recurrence rates and overall survival. There was one patient with bilateral breast cancer at presentation and this patient was included in the database twice (one entry for each cancer) for the purposes of the statistical analysis in respect of local recurrence (total number of cancers, 93).

SPSS for windows (version 11.0) was used to analyse the database. Kaplan-Meier tables were used to identify correlations between local recurrence, regional recurrence, systemic recurrence and overall survival and tumour characteristics and treatment parameters.

## Results

A total of 82 patients (83 cancers) did not receive adjuvant radiotherapy to the breast (group A). Ten patients received adjuvant radiotherapy to the breast (group B). The median age was 75 (range: 70 – 87) years. There were 13, 41 and 39 cancers of Grade 1, 2, and 3, respectively.

Group A: There were seven (8.4%) local recurrences with a median follow up of 37 (range 6 – 142) months, translating to an annual recurrence rate of 2.4% per annum. The median time to local recurrence was 17 (range 3 – 96) months. Group B: There were no local recurrences in this group observed during the same period. However, the difference in the local recurrence rate between the two groups was not statistically significant. This was mainly due to the small size of the group, during the period of the study because adjuvant radiotherapy to the breast was implemented after the year 1999 in this age group. For the purpose of the study, group B has been excluded from further statistical analysis.

Group A: Fifty-three cancers were ER positive and 26 were ER negative. ER status was not recorded in four patients. Forty of the 53 ER positive patients were treated with adjuvant tamoxifen. The median size of the cancers was 19 mm (range 2 mm – 40 mm). Seventeen patients had had axillary surgery and of those, 11 were node negative.

**Figure 1:**
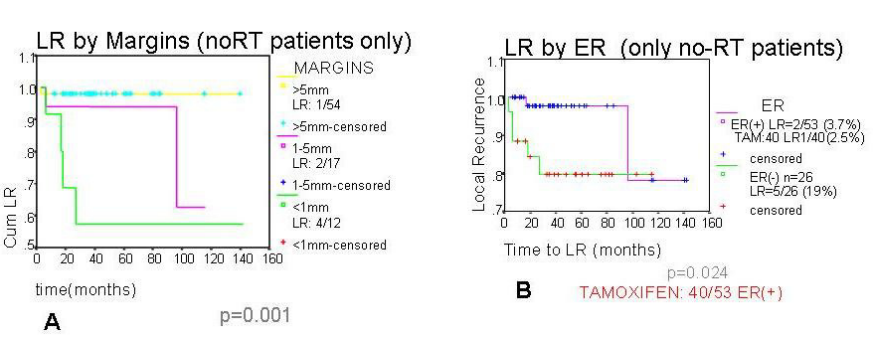
A) Patients who had no radiotherapy (RT) following breast conserving surgery – Relationship between local recurrence (LR) and margins of excision. B). Patients who had no radiotherapy (RT) following breast conserving surgery – Relationship between local recurrence (LR) and oestrogen receptor (ER) status.

Local recurrence in Group A significantly correlated with margins of excision (p < 0.001) (Figure 1a); there were 4/12, 2/17 and 1/54 local recurrences corresponding to margin status <1 mm, 1–5 mm and >5 mm, respectively. Local recurrence also correlated with ER status (p = 0.024) (Figure 1b) and size of tumour (p < 0.001). There were 2/53 recurrences in the ER positive group and 5/26 recurrences in the ER negative group. Within the ER positive group the local recurrence rate was 0.92% per annum (p.a.) (0.62% p.a. in patients treated with adjuvant tamoxifen, (1.78%p.a. when no adjuvant tamoxifen was given) (Table 1). There were 1/8, 1/35, 4/39 and 1/1 local recurrences corresponding to tumour size categories of <10 mm, 11–20 mm, 21–30 mm and 31–40 mm in size, respectively.

**Table 1:** Within the oestrogen receptor (ER) positive group, local recurrence (LR) was less than 1% per annum in patients treated with adjuvant tamoxifen.

N	LR(+)	LR(-)	
Adjvuant endocrine	1 (2.5%)	39	40
No adjuvant	1 (7.7%)	12	13
	2	52	53

p = 0.4 (non-significant)

**Figure 2:**
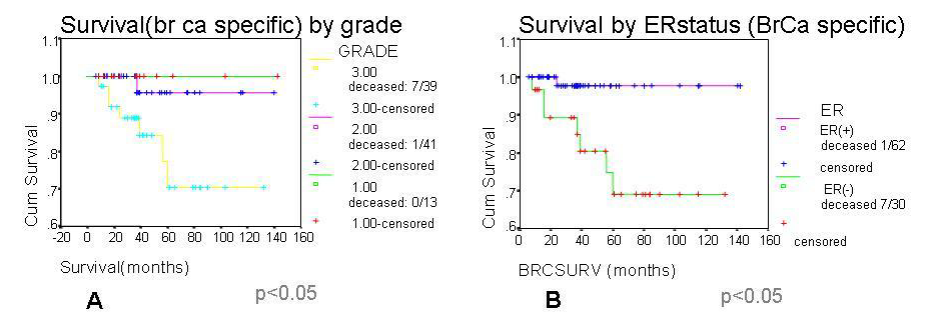
A) Breast cancer specific survival according to histological grade treated by breast conserving surgery B) Breast cancer specific survival according to oestrogen receptor (ER) status treated by breast conserving surgery.

Local recurrence did not correlate with lympho-vascular invasion (p = 0.7) or tumour grade (p = 0.5). However, histological grade correlated with regional (i.e. ipsilateral axillary lymph node) recurrence (p < 0.05) and overall survival (p < 0.005) (Figure 2). Survival also correlated with ER status (p < 0.01) (Figure 2).

## Discussion

Pooled data from individual studies or meta-analysis have shown that omission of radiotherapy after BCS generally produces adverse effects mainly on local recurrence and to some degree on survival [8-12]. The use of tamoxifen alone aiming to avoid radiotherapy has not been shown to produce satisfactory local control when 769 patients older than 50 years were investigated [13]. On the other hand, there are little data on which to assess the precise role of radiotherapy after BCS in the management of elderly patients with primary breast cancer. Data suggest that local recurrence rates are higher in younger women, with trials showing that they diminish with increasing age, especially when tamoxifen is given [14-18]; intact breast irradiation may not therefore be required to achieve similar local control in the elderly patients.

Before 1999, elderly patients (> 70 years) in Nottingham with primary breast cancer < 3 cm were given a choice between BCS and mastectomy, regardless of ER status. Patients were not given breast irradiation following successful wide local excision and this was the main group of patients studied in this paper (group A). Results show an excellent local control in the group of patients who had achieved adequate margins of excision (>5 mm at radial aspects) of an ER positive tumour and who were taking adjuvant tamoxifen (0.62% local recurrence rate per annum). This 0.62% per annum rate of local recurrence was noted regardless of margin width and tumour size, although these features were shown in this study to have correlation with local control in this group of patients. It would therefore be expected that local recurrence in the group of patients over the age of 70, treated by BCS and receiving tamoxifen for an ER positive tumour, would be even lower if the tumour was small (e.g. < 3 cm) and an adequate radial margin of excision (e.g. > 5 mm) was achieved. This is highly comparable to, or even better than, the local recurrence rate in younger patients who are treated with BCS followed by radiotherapy.

Similar results have been reported in other centres with both retrospective and prospective studies, showing reasonable local control in patients who received tamoxifen but no radiotherapy following lumpectomy [19,20]. In the retrospective study of Benhaim DI *et al., *[19] the results were better in lumpectomy with tamoxifen arm compared to standard treatment while in prospective study of Hughes KS *et al*., [20] there was no difference in 5 year overall survival though the local recurrence at 5 year was 3% higher. It would appear that omission of radiotherapy following BCS in elderly patients who are receiving adjuvant tamoxifen may be acceptable. We have since changed our treatment policy; elderly patients who have had successful wide local excision for an ER positive invasive breast carcinoma (with a circumferential margin = 5 mm) and who require adjuvant tamoxifen receive no radiotherapy to the intact breast. Patients with ER negative tumours are now routinely given radiotherapy. Results are being monitored and will be reported in the future.

## Competing interests

The author(s) declare that they have no competing interests.

## Authors' contributions

**KV **carried out data collection, statistical analysis and drafted the manuscript. **DALM**, **JFR **and **KLC **conceived of the study and participated in its design. SEP carried out the histological examination. **KLC **coordinated the whole study resulting in the completion of the final manuscript, which all authors read and approved.

## References

[B1] Arun C, Cheung KL (2003). Management of elderly primary breast cancer. Current medical literature. Breast Cancer.

[B2] August DA, Rea T, Sondak VK (1994). Age-related differences in breast cancer treatment. Ann Surg Oncol.

[B3] Merchant TE, McCormick B, Yahalom J, Borgen P (1996). The influence of older age on breast cancer treatment decisions and outcome. Int J Radiat Oncol Biol Phys.

[B4] Ballard-Barbash R, Potosky AL, Harlan LC, Nayfield SG, Kessler LG (1996). Factors associated with surgical and radiation therapy for early stage breast cancer in older women. J Natl Cancer Inst.

[B5] Hillner BE, Penberthy L, Desch CE, McDonald MK, Smith TJ, Retchin SM (1996). Variation in staging and treatment of local and regional breast cancer in the elderly. Breast Cancer Res Treat.

[B6] Hebert-Croteau N, Brisson J, Latreille J, Blanchette C, Deschenes L (1999). Compliance with consensus recommendations for the treatment of early stage breast carcinoma in elderly women. Cancer.

[B7] Clark GM, Osborne CK, McGuire WL (1984). Correlations between estrogen receptor, progesterone receptor, and patient characteristics in human breast cancer. J Clin Oncol.

[B8] Kantorowitz DA, Poulter CA, Rubin P, Patterson E, Sobel SH, Sischy B, Dvoretsky PM, Michalak WA, Doane K (1989). Treatment of breast cancer with segmental mastectomy alone or segemental mastectomy plus radiation. Radiother Oncol.

[B9] Early Breast Cancer Trialists' Collaborative Group (2000). Favourable and unfavourable effects on long-term survival of radiotherapy for early breast cancer: an overview of the randomised trials. Lancet.

[B10] Vinh-Hung V, Voordeckers M, Van de Steene J, Soete G, Lamote J, Storme G (2003). Omission of radiotherapy after breast-conserving surgery: survival impact and time trends. Radiother Oncol.

[B11] Vinh-Hung V, Verschraegen C (2004). Breast-conserving surgery with or without radiotherapy: pooled-analysis for risks of ipsilateral breast tumour recurrence and mortality. J Natl Cancer Inst.

[B12] Truong PT, Bernstein V, Lesperance M, Speers CH, Olivotto IA (2006). Radiotherapy omission after breast-conserving surgery is associated with reduced breast cancer-specific survival in elderly women with breast cancer. Am J Surg.

[B13] Fyles AW, McCready DR, Manchul LA, Trudeau ME, Merante P, Pintilie M, Weir LM, Olivotto IA (2004). Tamoxifen with or without breast irradiation in women 50 years of age or older with early breast cancer. N Engl J Med.

[B14] Gruenberger T, Gorlitzer M, Soliman T, Rudas M, Mittlboeck M, Gnant M, Reiner A, Teleky B, Seitz W, Jakesz R (1998). It is possible to omit postoperative irradiation in a highly selected group of elderly breast cancer patients. Breast Cancer Res Treat.

[B15] Sader C, Ingram D, Hastrich D (1999). Management of breast cancer in the elderly by complete local excision and tamoxifen alone. Aust N Z J Surg.

[B16] McCready DR, Chapman JA, Hanna WM, Kahn HJ, Yap K, Fish EB, Lickley HL (2000). Factors associated with local breast cancer recurrence after lumpectomy alone: postmenopausal women. Ann Surg Oncol.

[B17] de Csepel J, Tartter PI, Gajdos C (2000). When not to give radiation therapy after breast conservation surgery for breast cancer. J Surg Oncol.

[B18] Bartelink H, Horiot JC, Poortmans P, Struikmans H, Van den Bogaert W, Barillot I, Fourquet A, Borger J, Jager J, Hoogenraad W, Collette L, Pierart M (2001). Recurrence rates after treatment of breast cancer with standard radiotherapy with or without additional radiation. N Engl J Med.

[B19] Benhaim DI, Lopchinsky R, Tartter PI (2000). Lumpectomy with tamoxifen as primary treatment for elderly women with early-stage breast cancer. Am J Surg.

[B20] Hughes KS, Schnaper LA, Berry D, Cirrincione C, McCormick B, Shank B, Wheeler J, Champion LA, Smith TJ, Smith BL, Shapiro C, Muss HB, Winer E, Hudis C, Wood W, Sugarbaker D, Henderson IC, Norton L (2004). Lumpectomy plus tamoxifen with or without irradiation in women 70 years of age or older with early breast cancer. N Engl J Med.

